# Activation of VGluT2‐expressing neurons in the bed nuclei of the stria terminalis produces mouse manic‐like behaviors

**DOI:** 10.1111/cns.13537

**Published:** 2020-12-27

**Authors:** Ting‐Ting Liu, Wei Lin, Yu‐Qiu Zhang

**Affiliations:** ^1^ State Key Laboratory of Medical Neurobiology and MOE Frontiers Center for Brain Science Department of Translational Neuroscience Jing'an District Centre Hospital of Shanghai Institutes of Brain Science Fudan University Shanghai China; ^2^ Liao Ning Province Hospital Liaoning China

**Keywords:** glutamate, mania, mania‐like behavior, VGluT2

Mania, a state of pathologically elevated mood, energy, and activity, is the defining feature of bipolar disorder (BD). Evidences from animal studies suggest that an extended network including the medial prefrontal cortex, striatal, thalamic, and basal forebrain structures mediates mood disorder.^1^ The bed nuclei of the stria terminalis (BNST) is located in the basal forebrain, as key to psychiatric disorders, which is a center of integration information from limbic.^2^ A clinic study shows that deep brain stimulation of the BNST can improve resistant depression.^3^ BNST is dense with gamma‐aminobutyric acid (GABA) neurons which could induce anxiety after chemogenetic activation.^4^ Interestingly, there are a few glutamatergic neurons in the BNST with unknown functions. We, therefore, observed the effects of activating BNST neurons expressing vesicular glutamate transporters (VGluT2) by photogenetic manipulation. VGluT2‐IRES‐cre (JAX#016963) mice were used in this study. All animal experiments were approved by Committee on the Use of the Animals Experiments of Fudan (Permit No. SYXK 2009‐0082). The data are presented as the mean ± *SEM* and analyzed using GraphPad Prism 7.0 software (San Diego, CA, USA). All data from different groups were verified for normality and homogeneity of variance using Kolmogorov‐Smirnov and Brown‐Forsythe tests before analysis. Behavioral data were analyzed using one‐way ANOVA followed by post hoc Dunnett's test (more than 2 groups). All the hypothesis tests were 2‐tailed with *p* value less than 0.5 considered statistically significant. To examine the effects of activating VGluT2 neurons in the BNST on anxiodepressive or mania‐like behaviors, Cre‐dependent adeno‐associated virus (AAV) expressing channelrhodopsin ChR2 or mCherry (150 nl per hemisphere, OBiO Technology Co., Ltd. Shanghai) was stereotaxically injected by a Nanoliter 2010 Injector (WPI, Sarasota, Florida, USA) into the bilateral BNST (+0.23 AP, ±0.85 ML, −4.25 DV) in VGluT2‐IRES‐cre mice, and optical fibers were implanted at an angle of 10 degree, 0.3 mm above the virus injection sites (+0.23 AP, ±1.56 ML, −4.10 DV). Open field (OF) and tail suspension (TS) tests were performed at 2 weeks after optical fiber implantation (Figure 1A,B). For OF test, mice were gently placed into the center of an open box (40 cm × 40 cm × 30 cm) and allowed to explore for 3 min, and light (473 nm, 6–9 mW, 5 or 10 Hz, 25 ms) was subsequently delivered with 3‐min light off‐on‐off paradigm by a blue light laser (Thinker Tech Nanjing, China) and shut‐off for the next 3 min. For TS test, mice were suspended in the middle of a suspension box (30 cm × 30 cm × 30 cm) with the tape at 0.1 cm proximal to the tail tip. The distance between the mouse's nose and the apparatus floor was 2–3 cm. Mice were tested for 6 min, and the freezing time in the last 4 min was counted. To exclude influence of optical stimulation, we used wild‐type mice following the similar operation of virus injection and fiber implantation and filmed their behaviors and VGluT2‐IRES‐cre mice during 6‐min TS test with light on (473 nm, 6–9 mW, 5 Hz, 25 ms) or without light. The last 4‐min TS behaviors were analyzed. The animal behaviors were recorded and analyzed by video tracking system (EthoVision XT v11.5, Noldus BV). Activation of VGluT2 positive neurons by optogenetic stimulation was verified in the BNST slice recordings (Figure 1C,D). In a sequential 9‐min OF test, optogenetic activation of BNST VGluT2‐positive neurons strongly increased total travel distance (Figure 1F), velocity (Figure 1G), and number of jumping (Figure 1H) in open field (Figure 1E‐H, Movie S1, one‐way ANOVA, *p* < 0.01, *n* = 7). In contrast, photostimulation of BNST VGluT2 neurons expressing mCherry (*n* = 6) at different frequency did not change total travel distance (Supplementary S1B), velocity (Supplementary S1C), number of jumping (Supplementary S1D), and center time (Supplementary S1E) in the OF test (Supplementary S1A‐E). In TS test, wild‐type mice (*n* = 5) were not altered their behaviors during blue light activation, meanwhile, while VGluT2‐IRES‐cre mice (*n* = 7) almost kept struggling (Figure 1I, Movie S2). Furthermore, we tried to observe the behaviors of mice in elevated plus maze (EPM) test. Optogenetic activation of the VGluT2‐IRES‐cre mice in ChR2 mCherry group led to improved velocity (Figure 2). We also observed the risk‐taking behaviors of mice in the same group appearing frequently jumping to the floor from the open arm of EPM (Figure 2). Collectively, these results indicate that activation of VGluT2‐positive excitatory neurons in the BNST produces hyperactivity and promotes risk‐taking behaviors in mice.

**FIGURE 1:**
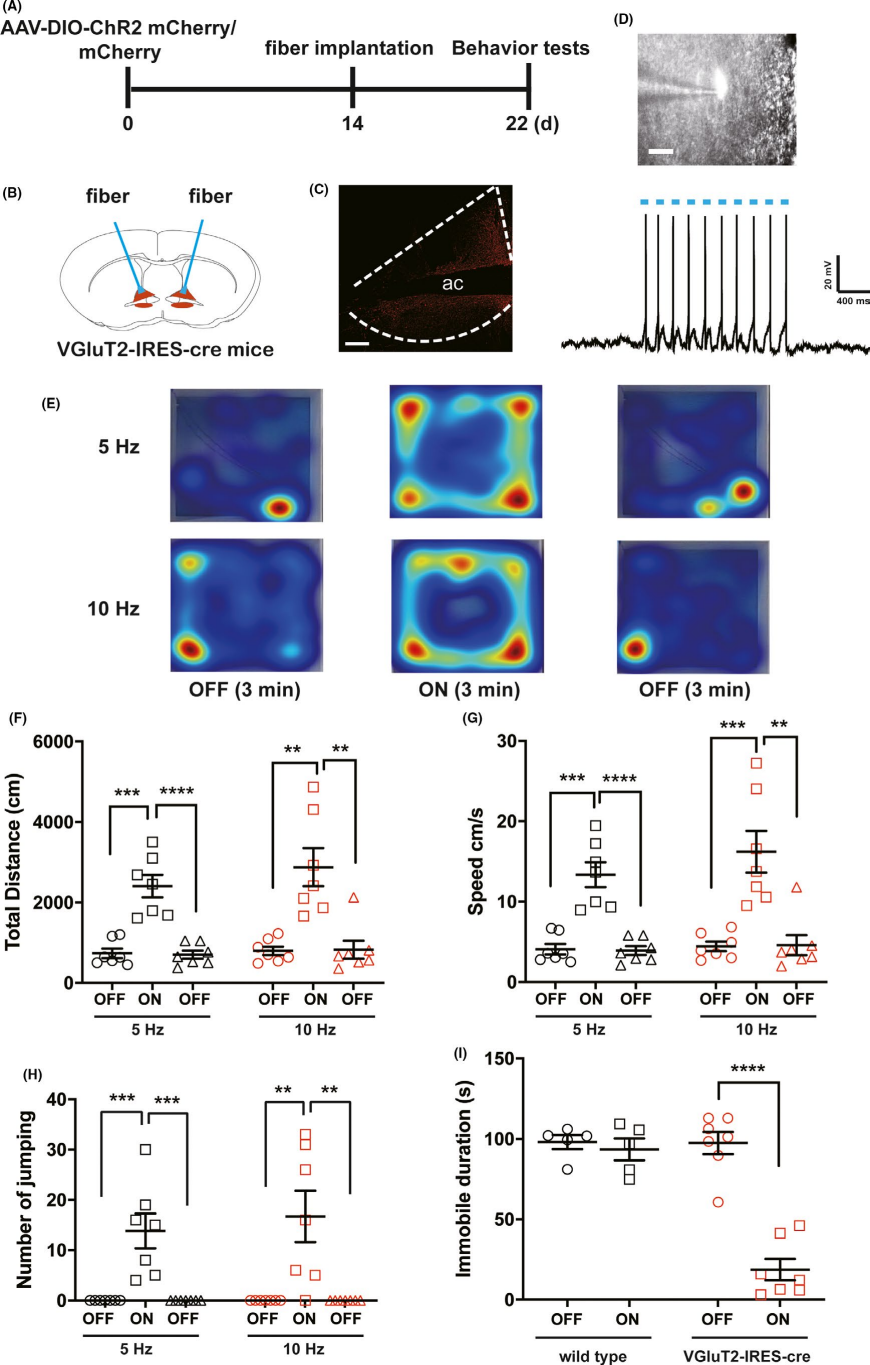
Optogenetic activation of BNST VGluT2 neurons induces manic‐like behaviors

**FIGURE 2:**
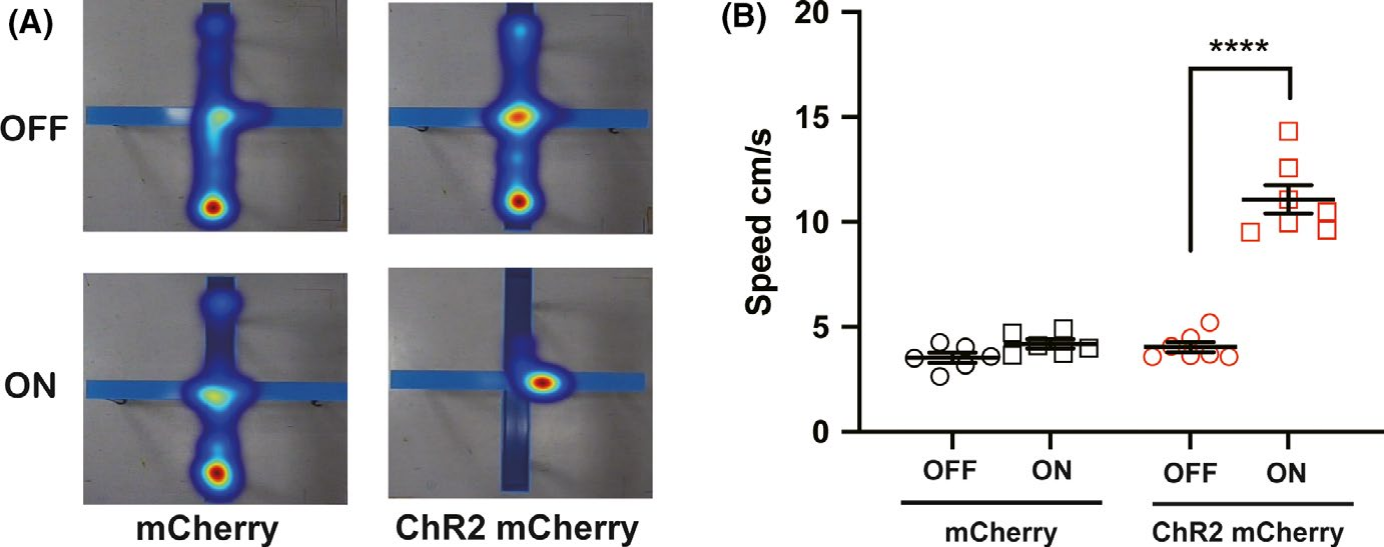
Optogenetic activation of VGluT2 + neurons in the BNST improved velocity and produced risk‐taking behaviors during elevated plus maze (EPM)

Early evidence from depressive patients and rodent models showed that neurons atrophied and limbic brain regions and network function were abnormal in cortical and limbic brain regions.^5^ The BNST, as a part of limbic brain, was also confirmed an increased activation during threat monitoring in patients with anxiety disorder.^6^ Another report verified that deep brain stimulation (DBS) of BNST in the five depressive patients improved their clinical symptoms.^3^ The BNST is comprised of many distinct subnuclei and it is understandable that different subnuclei have separated afferent and efferent projections and plays various functions.^7^ Mazzone et al^4^ demonstrated that chemogenetic activation of BNST Vgat‐positive neurons significantly increased anxiety‐like behavior. Recently, human KCTD gene family has been implicated in neuropsychiatric disorders, including depression and schizophrenia. As an auxiliary submit of the GABA_B1/2_ complex, KCTD12 was involved in bipolar I disorder, depression and schizophrenia. Similarly, kctd12‐knockout mice exhibit emotional related phenotypes. Another KCTD family member, KCTD13, was also reported to contribute to autism and schizophrenia.^8^ In the present study, using a genetic marker to label BNST VGluT2 neurons, we found that activation of BNST excitatory neurons directly produces hyperactivity, an appearance of behavior similar to animal model of mania.^9^ These results further support the hypothesis that the unbalance in excitatory and/or inhibitory neurotransmitters caused psychological illnesses.^10^ VGluT2‐positive neurons in the BNST, which are often neglected,^2^ may promote manic behaviors in normal mice, indicating a potential direction of understanding of BD in the future.

## CONFLICT OF INTEREST

The authors declare no conflict of interest.

## Supporting information

Fig S1

Movie S1

Movie S2
