# Supplementary material for: Activation of VGluT2‐expressing neurons in the bed nuclei of the stria terminalis produces mouse manic‐like behaviors
Source: CNS Neurosci Ther. 2020 Dec 27;27(2):259–62. doi: 10.1111/cns.13537 (PMC7816202; doi:10.1111/cns.13537)
Supplement: Supplementary file 1 — Fig S1 [file CNS-27-259-s001.docx]

**Supplemental Information**

**Activation of VGluT2-expressing neurons in the bed nuclei of the stria terminalis produces mouse manic-like behaviors**

Ting-ting Liu^1, 2^ ·Wei Lin^1^· Yu-Qiu Zhang^1^

^1^State Key Laboratory of Medical Neurobiology and MOE Frontiers Center for Brain Science, Department of Translational Neuroscience, Jing’an District Centre Hospital of Shanghai, Institutes of Brain Science, Institues of Integrative Medicine, Fudan University, Shanghai 200032, China.

^2^ Liao Ning Province Hospital, Liaoning 110000, China.

**Inventory of Supplemental Information**

**Supplementary 1** Optogenetic activation of VGluT2+ neurons expressing mCherry

within the BNST do not affect behaviors in the 9-min sequential OF test.

**Figure S1.** related to Figure 1, Optogenetic activation of VGluT2+ neurons in the BNST improved velocity and produce risk-taking behaviors during elevated plus maze (EPM) test.

**Movi S1.** related to Figure 1, Optogenetic activation of the BNST VGluT2+ neurons promote manic-like behaviors during open filed (OF) test. Blue light (473 nm, 6 mW, 25ms, 5Hz or 10Hz) stimulation of the bilateral BNST induces hyperactivity a VGluT2-IRES-cre mouse expressing ChR2.

**Movie S2.** related to Figure 1, Optogenetic activation of the BNST VGluT2+ neurons increase struggling behaviors in the tail suspension (TS) test. VGluT2-IRES-cre mouse expressing ChR2 keeps struggling during TS test following blue light stimulation (473 nm, 6-9 mW, 5Hz, 25ms).


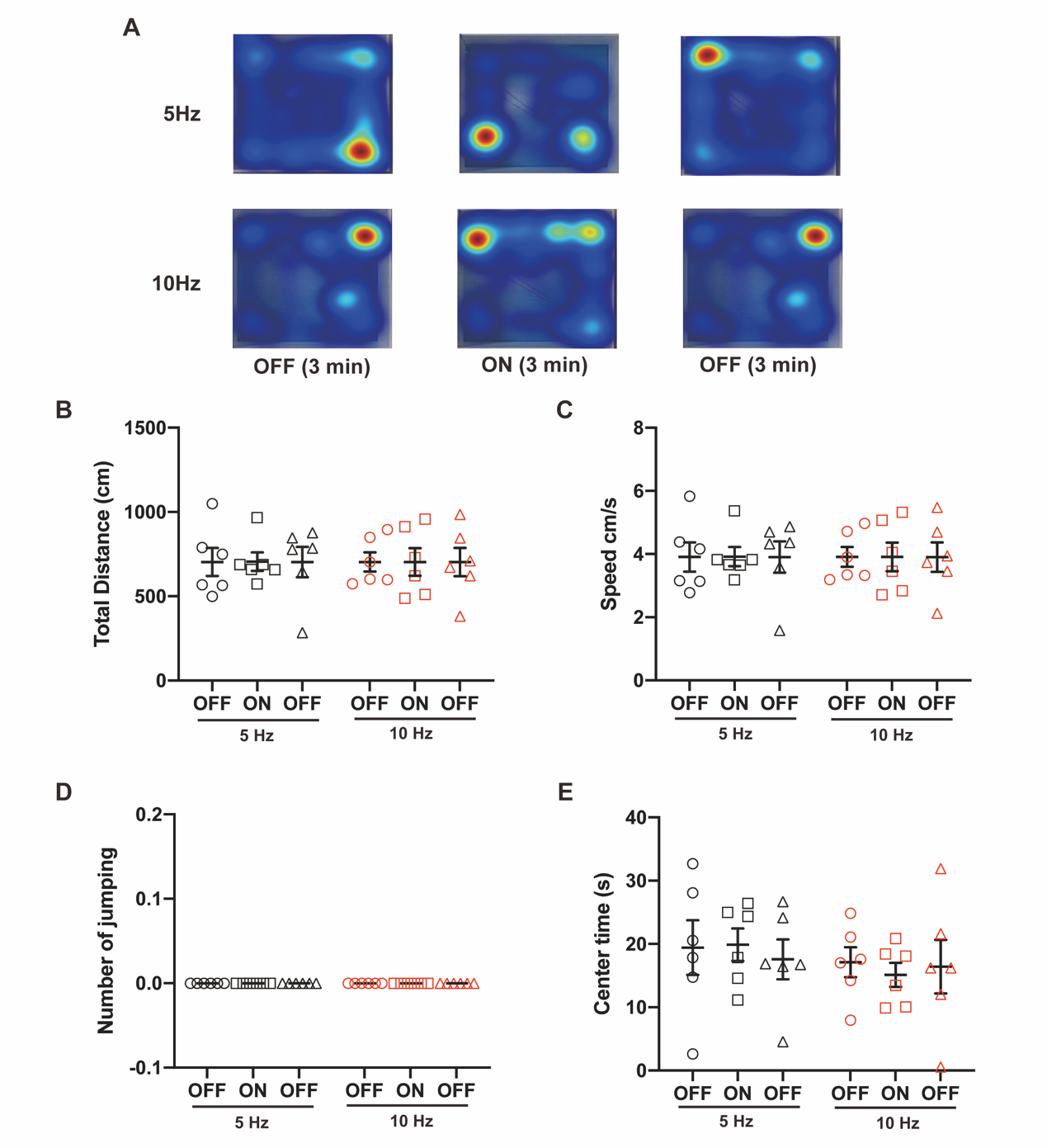


**Supplementary 1** Optogenetic activation of VGluT2+ neurons expressing mCherry within the BNST do not affect behaviors in the 9-min sequential OF test.

**A.** The heat traces of VGluT2-cre mice by bilateral activation of VGluT2+ neurons expressing mCherry within the BNST during open field (OF) test by blue light (5Hz and 10Hz) stimulation with 3 min light-off, 3 min light-on and 3 min light-off. **B-E.** Photostimulation of BNST VGluT2 neurons expressing mCherry (n=6) by blue light (5Hz and 10Hz) did not affect total travel distance (B), velocity (C), number of jumping (D) and center time (E) in 9-min sequential open field test. one-way ANOVA, n=6. Data are mean ± s.e.m.
